# Non-coding RNA regulation of Magang geese skeletal muscle maturation via the MAPK signaling pathway

**DOI:** 10.3389/fphys.2023.1331974

**Published:** 2024-01-19

**Authors:** Longsheng Hong, Danning Xu, Wanyan Li, Yifeng Wang, Nan Cao, Xinliang Fu, Yunbo Tian, Yugu Li, Bingxin Li

**Affiliations:** ^1^ College of Animal Science and Technology, Zhongkai University of Agriculture and Engineering, Guangzhou, China; ^2^ College of Veterinary Medicine, South China Agricultural University, Guangzhou, China; ^3^ College of Computer Science and Software Engineering, Shenzhen University, Shenzhen, China

**Keywords:** Magang goose, myofiber maturation, ceRNA, skeletal muscle, MAPK signaling pathway

## Abstract

Skeletal muscle is a critical component of goose meat and a significant economic trait of geese. The regulatory roles of miRNAs and lncRNAs in the maturation stage of goose skeletal muscle are still unclear. Therefore, this study conducted experiments on the leg muscles of Magang geese at two stages: 3-day post-hatch (P3) and 3 months (M3). Morphological observations revealed that from P3 to M3, muscle fibers mainly underwent hypertrophy and maturation. The muscle fibers became thicker, nuclear density decreased, and nuclei moved towards the fiber edges. Additionally, this study analyzed the expression profiles of lncRNAs, miRNAs, and mRNAs during the skeletal muscle fiber maturation stage, identifying 1,949 differentially expressed mRNAs (DEMs), 21 differentially expressed miRNAs (DEMIs), and 172 differentially expressed lncRNAs (DELs). Furthermore, we performed enrichment analyses on DEMs, cis-regulatory genes of DELs, and target DEMs of DEMIs, revealing significant enrichment of signaling pathways including MAPK, PPAR, and mTOR signaling pathways. Among these, the MAPK signaling pathway was the only pathway enriched across all three types of differentially expressed RNAs, indicating its potentially more significant role in skeletal muscle maturation. Finally, this study integrated the targeting relationships between DELs, DEMs, and DEMIs from these two stages to construct a ceRNA regulatory network. These findings unveil the potential functions and mechanisms of lncRNAs and miRNAs in the growth and development of goose skeletal muscle and provide valuable references for further exploration of the mechanism underlying the maturation of Magang geese leg muscle.

## 1 Introduction

Skeletal muscle is an important part of an animal’s body, accounting for almost half of its total body weight. It is the main source of meat protein and also directly affects an important economic trait, namely meat production (Ye et al., 2016; Zaukuu et al., 2021). The genesis and development of poultry skeletal muscle mainly include the embryonic stage and the post-hatching stage. During the embryonic period, the myogenic cells undergo a series of proliferation, migration, and differentiation to form myocytes. Myoblasts proliferate further and differentiate into muscle tubes, which eventually differentiate into muscle fibers (Picard et al., 2002; Zaukuu et al., 2021). After hatching, the growth of skeletal muscle is mainly achieved through the hypertrophy of muscle fibers. This process is accompanied by the conversion of proteins and the activation of muscle satellite cells. Proteins undergo a transformation process involving synthesis, degradation, and repair ability, providing the necessary building and repair materials for muscle fibers. At the same time, muscle satellite cells are activated, undergo proliferation, and subsequently differentiate into muscle cells, ultimately fusing into mature myotubes. In addition to the intricate cellular development during muscle fiber hypertrophy, the development of skeletal muscle also depends on the precise regulation of multiple myogenic genes (Rehfeldt et al., 2000; Shahjahan, 2015).

Poultry is currently the largest animal source of global meat production, and the OECD-FAO forecasts that poultry will account for more than half of the increase in global meat production over the next decade (OECD/FAO, 2022). Compared with other poultry, goose meat has characteristics such as high-quality protein, low cholesterol, and a high concentration of polyunsaturated fatty acids (Guo et al., 2020). China, as the world’s largest consumer of goose meat, consumed at least 4.29 million tons in 2021, while its popularity continues to grow in Asia and Europe, according to statistics from the Food and Agriculture Organization (FAO) (https://www.fao.org/faostat/zh/#data/QCL). Additionally, consumers are placing greater emphasis on healthy eating habits, leading to an increasing demand for goose meat. Magang geese exhibit excellent growth performance and meat quality, with an annual yield close to one-tenth of national production (Li et al., 2021). Therefore, exploring the molecular mechanism behind skeletal muscle maturation in Magang geese holds significant economic value in meeting the demand for goose meat while improving its yield and quality.

Non-coding RNAs (ncRNAs) are a class of nucleotides that do not encode proteins but can regulate cell physiology and shape cell function (Panni et al., 2020). Based on their length, function, and structural characteristics, ncRNAs can be divided into microRNAs (miRNAs), long non-coding RNAs (lncRNAs), circular RNAs (circRNAs), and others (Hombach and Kretz, 2016). Currently, an increasing number of studies have demonstrated the close relationship between miRNAs and lncRNAs in the development process of poultry skeletal muscle, including myoblast proliferation, differentiation, apoptosis, as well as myofiber maturation and hypertrophy (Li et al., 2016; Shi et al., 2022). Additionally, more attention has been paid to the competing endogenous RNA (ceRNA) hypothesis. This hypothesis suggests that mRNAs and lncRNAs with the same microRNA response elements can competitively bind to miRNAs as ceRNAs. As a result, they regulate the degradation or translation inhibition of target mRNAs (Salmena et al., 2011; Tay et al., 2014). The ceRNA hypothesis has also become mainstream in lncRNA research by revealing the role of lncRNAs in skeletal muscle growth and development through constructing ceRNA networks (Zhang et al., 2021a). Furthermore, there are also studies demonstrating how a single lncRNA acts as a ceRNA to regulate the growth and development of poultry skeletal muscle (Li et al., 2019). However, the expression profile and related functions of ncRNAs in skeletal muscle growth and development in geese remain unknown.

In this study, we selected leg muscles of Magang geese at 3-day post-hatch (P3) and 3 months of age (M3) for experiments. HE staining was performed on the collected skeletal muscles to investigate the tissue structure and morphology of Magang geese at different growth stages. Subsequently, RNA sequencing libraries were constructed to comprehensively analyze the expression profiles of lncRNAs, miRNAs, and mRNAs during the maturation stage of the Magang goose skeletal muscle. Furthermore, functional enrichment analysis was conducted on differentially expressed mRNAs (DEMs), differentially expressed miRNAs (DEMIs), and differentially expressed lncRNAs (DELs) at various stages. A comprehensive ceRNA network was then constructed to further explore the regulatory mechanisms involved in skeletal maturation. This study provides a systematic investigation and identification of the expression profiles of lncRNAs, miRNAs, and mRNAs during the maturation stage of goose leg skeletal muscle for the first time, offering insights into potential roles played by miRNAs and lncRNAs in growth and development.

## 2 Materials and methods

### 2.1 Animals and sample collection

In this study, 12 female Magang geese at different stages of growth (P3 and M3) were used. After the slaughter and evaluation of experimental procedures for all Magang geese, which were approved by the Animal Ethics Committee of Zhongkai University of Agriculture and Engineering (Approval Code: 2022110801), six Magang geese were randomly selected for euthanasia in each period. Subsequently, the leg skeletal muscle was aseptically collected and divided into three parts. Two parts were immediately stored in liquid nitrogen for RNA seq and qRT-PCR analysis, while the other part was fixed in 4% paraformaldehyde for histological observation. The gender of the Magang geese was identified using PCR amplification of chromodomain helicase DNA binding protein 1 (*CHD1*), with primer sequences shown in Supplementary Table S1.

### 2.2 Histological observation of the Magang geese skeletal muscle

The collected leg muscle of Magang geese was fixed in 4% paraformaldehyde for 48 h, then dehydrated, embedded in paraffin, cut into 5 μm sections, and stained with HE staining (*n* = 3). Finally, the microscopic structure of the leg muscle was observed by using an optical microscope (OLYMPUS, Tokyo, Japan) and CaseViewer software (V 2.4.0).

### 2.3 RNA extraction, library construction, and illumina sequencing

In this study, three long RNA libraries and three miRNA libraries (*n* = 3) were constructed separately from the mature stage of the leg muscles of Magang geese (P3 and M3). Total RNA from Magang goose leg muscle was isolated and purified using Trizol reagent (Invitrogen, Carlsbad, CA, United States). The purified total RNA was then assessed using NanoDrop ND-1000 (NanoDrop, Wilmington, DE, United States) and Bioanalyzer 2100 (Agilent, CA, United States) to ensure that it met the following criteria for downstream experiments: concentration > 50 ng/μL, RNA integrity number value > 7.0, and OD 260/280 > 1.8. Subsequently, three samples were used from each age group for RNA-Seq. The ribosomal RNA was removed using a Ribo-Zero Gold rRNA Removal Kit (Illumina, San Diego, United States) with 10 ug of total RNA. The RNA was then segmented, reverse-transcribed, spliced, and PCR amplified to construct a cDNA library. The constructed cDNA libraries were sent to LC-BIO Bio-tech Ltd for double-ended sequencing on Illumina HiSeq 6000, and the sequencing mode was PE150. In addition, TruSeq Small RNA Sample Prep Kits (Illumina, San Diego, United States) were used to prepare small RNA sequencing library. After the library was prepared, it was single-end sequenced by Illumina Hiseq 2500, and the sequencing read length was 1 × 50 bp.

### 2.4 Validation of RNA-seq data by qRT-PCR

For mRNA and lncRNA, RNA with an A260/280 ratio of between 1.8 and 2.1 and A260/230 ratio of >2.0 was reverse transcribed into cDNA by the TaKaRa reverse transcription kit (RR036A, Takara, Tokyo, Japan). For miRNA, the Bulge-Loop miRNA qRT-PCR Starter Kit (Ribobio Technology, Guangzhou, China) was used to transcribe RNA without genomic DNA into cDNA using stem-loop RT primers. Furthermore, the SYBR PreMix Ex TaqTM II (TliRNaseH Plus) kit (Thermo Fisher, San Jose, CA, United States) was used for real-time fluorescence quantification, which was performed using the ABI QuantStdio7 real-time PCR instrument with three technical replicates for each sample. For mRNA and lncRNA, *GAPDH* was used as the reference gene, while for miRNA, U6 snRNA was used as the reference gene. The primer sequences are shown in Supplementary Table S1. The relative expression levels of each RNAs were calculated using the 2^−ΔΔCT^ method, and statistical analysis and graphs were generated using GraphPad Prism 7.00 software (Prism, San Diego, CA, United States).

### 2.5 Differential expression mRNAs screening and enrichment analysis

After sequencing, Cutadapt (V 4.2) and FastQC (V 0.11.9) were employed for the filtration and validation of the acquired high-quality clean data (Martin, 2011; Thompson et al., 2020). Subsequently, these high-quality clean data were aligned to the Ansers cygnoides reference genome (https://www.ncbi.nlm.nih.gov=Anser+cygnoides) using HISAT2 (V 2.2.1), enabling mapping of the reads to the reference genome (Pertea et al., 2016). The mapped reads for each sample were assembled utilizing StringTie (V 2.1.6), followed by comparison and reconstruction of the assembled transcripts into a composite transcriptome using gffcompare software (V 0.9.8). Once the synthesized transcriptome was generated, expression levels of the transcripts were evaluated through StringTie and ballgown (V 2.30.0), subsequently calculating FPKM values (fragments per kilobase of exon per million fragments mapped) (Kovaka et al., 2019).

Based on FPKM, mRNAs with *p* < 0.001 and |log_2_(Fold change)| ≥ 1 were defined as DEMs using DESeq2 software (v1.10.1) (Love et al., 2014). To explore the potential functions of DEMs, we used the Gene Ontology online website (http://geneontology.org/) for conducting Gene Ontology (GO) analysis (Consortium, 2021). Furthermore, we also utilized the KOBAS online website (http://kobas.cbi.pku.edu.cn/) to perform Kyoto Encyclopedia of Genes and Genomes (KEGG) pathway analysis on the DEMs (Bu et al., 2021). The GO terms and KEGG signal pathways with a *p*-value less than 0.05 indicate significant enrichment.

### 2.6 Differential expression miRNA screening and gene enrichment analysis

ACGT101-miR software (LC Sciences, Houston, Texas, United States) was used to remove 3′ joints and junk sequences in order to obtain clean data, retain sequences with base lengths of 18–26 nt, aligning and filtering RNA families (rRNA, tRNA, snRNA, snoRNA) and repeat sequences in order to obtain high-quality sequences. Subsequently, miRNA identification was performed by comparing the precursor and genome sequences to obtain valid data for subsequent analysis. Next, DEMIs were filtered based on the criteria of *p* < 0.001 and |log_2_(Fold change)| ≥ 1. TargetScan (V 7.1) and miRanda (V 3.3a) were used to predict the DEMs targeted by DEMIs. The predicted DEMs with a TargetScan context score > 80 and a miRanda energy < −20 were considered to have targeting relationships with the DEMIs. Subsequently, the DEMI-DEM network was constructed using the cytoscape (V 3.9.1). In addition, GO and KEGG enrichment analysis of DEMIs targeted genes were performed using Gene Ontology and KOBAS online website, respectively.

### 2.7 Differential expression lncRNA screening and gene enrichment analysis

After filtering, screening, aligning, and identifying the reconstructed transcriptome, transcripts that overlapped with known mRNAs, lncRNAs, or those shorter than 200 bp were filtered out. Then, the coding potential of new transcripts was predicted using default parameters of CPC 2.0 (http://cpc2.gao-lab.org/) and CNCI 2.0 (https://github.com/www-bioinfo-org/CNCI#install-cnci). Transcripts with CPC scores less than 0.5 and CNCI scores less than zero were selected for analysis of potential lncRNAs with category codes (i, j, o, u, x). Similarly, the lncRNA comprehensive transcriptome was reconstructed using StringTie and GffCompare, with lncRNA transcript expression levels and FPKM values evaluated using StringTie and Ballgown. Subsequently, DELs were filtered using criteria of *p* < 0.001 and |log_2_(Fold change) | ≥ 1. To investigate the functions of DELs, the Python script was employed to filter for 100,000 coding genes upstream and downstream. Furthermore, the DELs’ cis-regulated genes were subjected to GO and KEGG enrichment analysis using the Gene Ontology and KOBAS online websites, respectively. TargetScan and miRanda were employed to predict the targeted DELs by DEMIs, where predicted DELs with TargetScan context scores greater than 80 and miRanda energies less than −20 were considered to define the targeting relationship with the DEMIs. Finally, DELs-DEMIs networks were constructed using Cytoscape.

### 2.8 Construction of the lncRNA-miRNA-mRNA regulatory network

Based on the targeted prediction network of DEMI-DEM and DEMI-DEL at different growth and development stages of Magang geese leg muscle, a ceRNA regulatory network was constructed using Cytoscape according to the ceRNA theory. In addition, we used the Centiscape plugin (V 2.2) to analyze the centrality-related indices of the ceRNA network, aiming to explore the structure and characteristics of the lncRNA-miRNA-mRNA network. Finally, we used Venny (V 2.1) to intersect the KEGG enrichment results of DEMs, cis-regulatory genes of DELs, and target DEMs of DEMIs.

## 3 Results

### 3.1 Histological characteristics of Magang geese skeletal muscle

**FIGURE 1:**
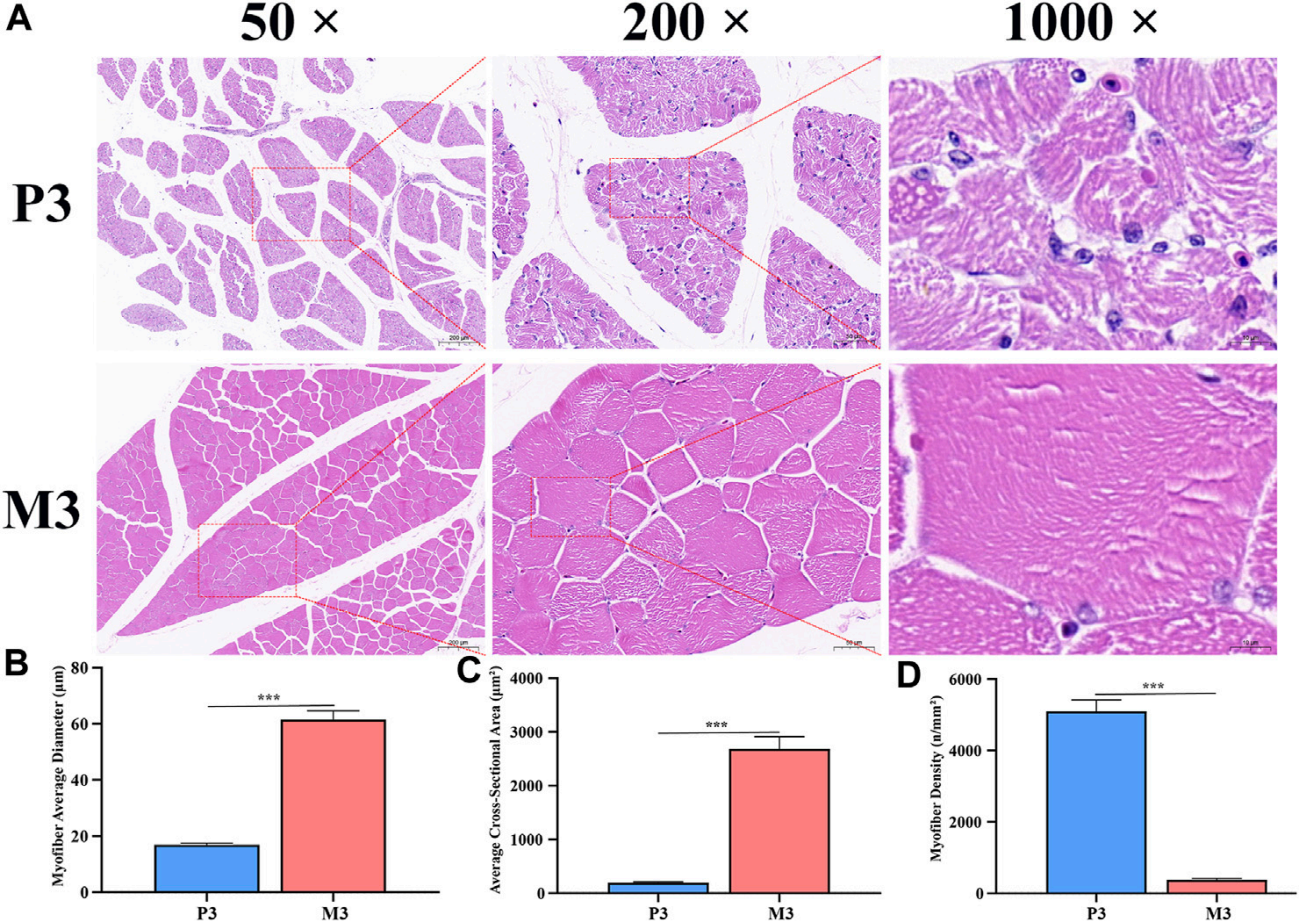
Histological characteristics of Magang goose leg skeletal muscle in post-hatch 3 days and 3 months of age. HE stained sections **(A)**. 50×, 200×, and 1000× respectively represent the magnification of the slide under an optical microscope by 50 times, 200 times, and 1000 times. Myofiber diameter **(B)**, myofiber cross-sectional area **(C)** and myofiber density **(D)** of leg skeletal muscle.

To gain a better understanding of the differential performance of skeletal muscle fibers at different stages, we performed HE staining on muscle slices from P3 and M3 samples to observe and analyze the morphology of the muscle fibers. At 3 days post-hatching, the skeletal muscle fibers of Magang geese exhibited relatively complete morphology, but their arrangement was relatively loose, with a higher number of cell nuclei in the cross-section. As the muscle fibers matured and hypertrophied, the arrangement of muscle fibers in the M3 skeletal muscle became denser. Concurrently, the muscle nuclei gradually shifted towards the periphery of muscle fibers, resulting in a decrease in the density of muscle nuclei (Figure 1A). The diameter and area of M3 group myofibers exhibited significant increases compared to those of the P3 group, with a diameter approximately 3.6 times larger and an area approximately 13 times greater than that of the P3 group (Figures 1B, C). In addition, as shown in Figure 1D, the density of myofibers significantly decreases with age.

### 3.2 Overview of RNA-seq and qRT-PCR validation

**FIGURE 2:**
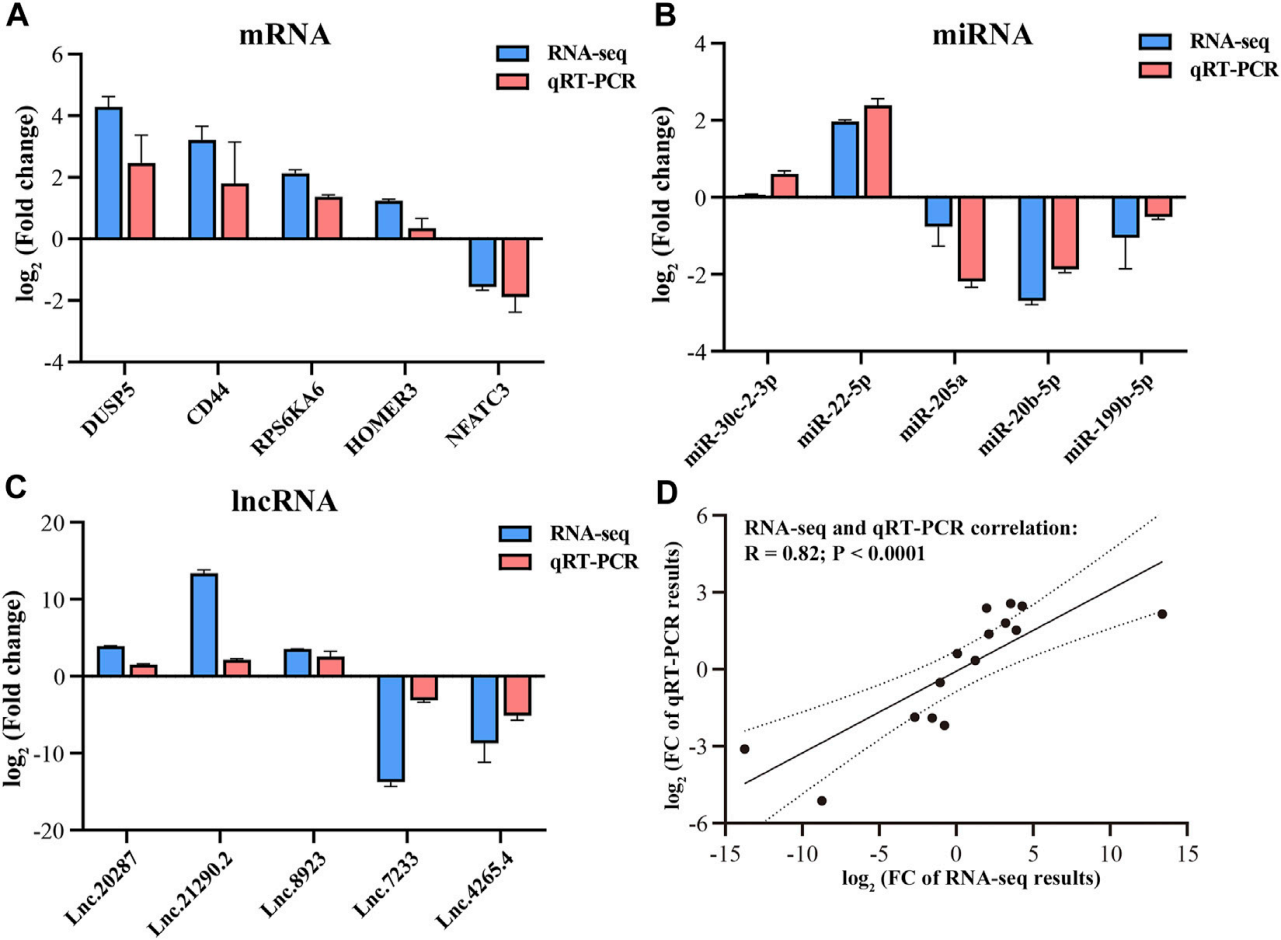
Validation of RNA-seq data by qRT-PCR. qRT-PCR verification for mRNAs **(A)**, miRNAs **(B)** and lncRNAs **(C)**. Correlation between RNA-seq and RT-qPCR results **(D)**.

The transcriptome data of Magang goose leg muscles in this study can be publicly accessed at the Sequence Read Archive (SRA) PRJNA989736 (https://www.ncbi.nlm.nih.gov/bioproject/). In the long-chain RNA libraries, a total of 542.34 million raw reads were obtained, with an average of 90.39 million raw reads per sample. After quality control, a total of 453.22 million (84%) reads were retained as clean reads, with an average of 75.53 million clean reads per sample library for subsequent analysis (Table 1). Each sample had Q20% and Q30% values exceeding 99.99 and 98.4, respectively, indicating high sequencing accuracy and reliability. Through alignment analysis, it was found that an average of 66.87 million (88.55%) clean reads could be successfully mapped to the Ansers cygnoides reference genome (Supplementary Table S2). In the miRNA libraries, a total of 64.47 million total raw reads were obtained, and after quality control, 42.35 million (66%) valid reads were obtained (Table 2). Among them, 5.71 million unique raw reads were identified, with an average of 0.37 million valid unique reads per sample. Furthermore, to validate the accuracy of the RNA-seq data, we randomly selected 5 mRNAs, 5 miRNAs, and 5 lncRNAs for qRT-PCR validation. The results demonstrated a significant correlation (Pearson correlation coefficient of 0.82, *p* < 0.0001) between qRT-PCR validation and RNA-seq data, thereby supporting the reliability of the RNA-seq data and confirming the effectiveness of subsequent analyses (Figure 2).

**TABLE 1:** Characteristics of the reads from long-chain RNA sequencing libraries.

Sample	Raw data	Valid data	Valid ratio (%)	Q20 (%)	Q30 (%)	GC content (%)
P3_1	100,932,184	81,800,100	81.04	99.99	98.22	46.00
P3_2	79,811,274	68,892,586	86.32	99.99	98.37	45.00
P3_3	84,644,806	72,358,742	85.49	99.99	98.39	45.00
M3_1	89,556,032	72,657,630	81.13	99.99	98.44	48.00
M3_2	80,895,710	67,003,172	82.83	99.99	98.49	47.00
M3_3	106,501,436	90,509,214	84.98	99.99	98.38	46.50

**TABLE 2:** Characteristics of the reads from miRNA sequencing libraries.

Sample	Total reads			Unique reads		
	Raw reads	Valid reads	Valid ratio (%)	Raw reads	Valid reads	Valid ratio (%)
P3_1	10,471,894	7,340,400	70.10	773,906	296,479	38.31
P3_2	10,165,032	6,390,488	62.87	1,697,353	733,552	43.22
P3_3	11,327,623	7,425,896	65.56	1,299,890	578,203	44.48
M3_1	11,117,180	7,270,847	65.40	659,886	201,387	30.52
M3_2	11,225,142	7,119,429	63.42	673,578	219,605	32.60
M3_3	10,158,271	6,804,112	66.98	608,813	190,007	31.21

### 3.3 Identification and potential function analysis of DEMs

**FIGURE 3:**
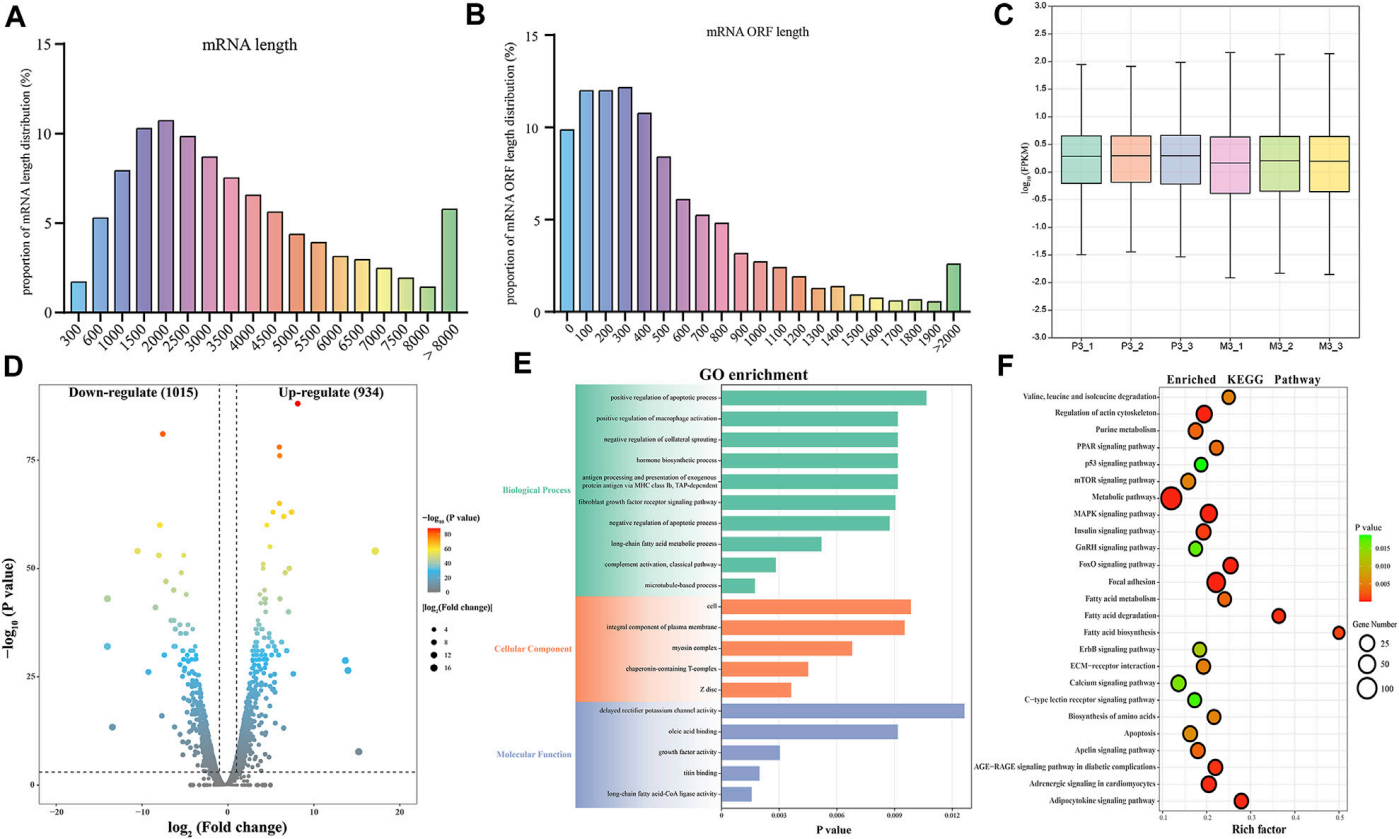
Identification and potential function analysis of DEMs. Proportion of mRNA length distribution **(A)** and mRNA ORF length distribution **(B)**. The boxplot of FPKM for mRNA **(C)**. Volcano plot of 1949 DEMs **(D)**. GO enrichment plot **(E)** and KEGG enrichment plot **(F)** of DEMs. The GO enrichment plot showed the top 10 most significant biological processes, the top 5 most significant cellular components, and the top 5 most significant molecular functions. The KEGG enrichment plot showed the top 25 most significant KEGG pathways.

In this study, we identified a total of 19,866 candidate mRNAs, primarily ranging in length from 1,000 to 3,500 bp (Figure 3A). The distribution of ORF lengths can provide important insights into gene structure and function. As shown in Figure 3B, the majority of mRNA ORF lengths in the gastrocnemius muscle of Magang geese were below 500 bp, accounting for approximately 65% of the total. Additionally, using FPKM to quantify transcript abundance, we found that the average expression level of mRNA in the skeletal muscle of 3-month-old Magang geese was lower than that in the P3 group (Figure 3C). To investigate potential regulatory mRNAs in the two groups of goose muscles, a total of 1,949 DEMs, including 1,015 downregulated mRNAs and 934 upregulated mRNAs (Figure 3D; Supplementary Table S3). Subsequently, GO and KEGG enrichment analyses were performed to explore the potential regulatory roles of these DEMs during skeletal muscle maturation.

The GO enrichment analysis revealed that the most enriched terms in the biological process category were microtubule-based processes, complement activation processes, long-chain fatty acid metabolic processes, negative regulation of apoptotic processes, and fibroblast growth factor receptor signaling pathways. In the cellular component category, the top three enriched terms were Z-disc, chaperonin-containing T-complex, and myosin complex. In the molecular function category, the most enriched terms were long-chain fatty acid-CoA ligase activity, titin binding, growth factor activity, and oleate binding (Figure 3E; Supplementary Table S4). In the KEGG enrichment analysis, the DEGs were found to be enriched in pathways related to cell growth and proliferation regulation, including the MAPK signaling pathway, FoxO signaling pathway, mTOR signaling pathway, p53 signaling pathway, and apoptosis. Among these, the MAPK signaling pathway exhibited the most significant enrichment. Pathways related to metabolism and energy regulation included the adipocytokine signaling pathway, fatty acid degradation, fatty acid biosynthesis, fatty acid metabolism, PPAR signaling pathway, and insulin signaling pathway. Furthermore, several signaling pathways associated with extracellular matrix connections and signal transduction, such as Focal adhesion, ECM-receptor interaction, and calcium signaling pathway, were also identified in the KEGG enrichment analysis (Figure 3F; Supplementary Table S5). The interaction of these signaling pathways regulates skeletal muscle maturation.

### 3.4 Identification and analysis of DEMIs associated with skeletal muscle maturation

**FIGURE 4:**
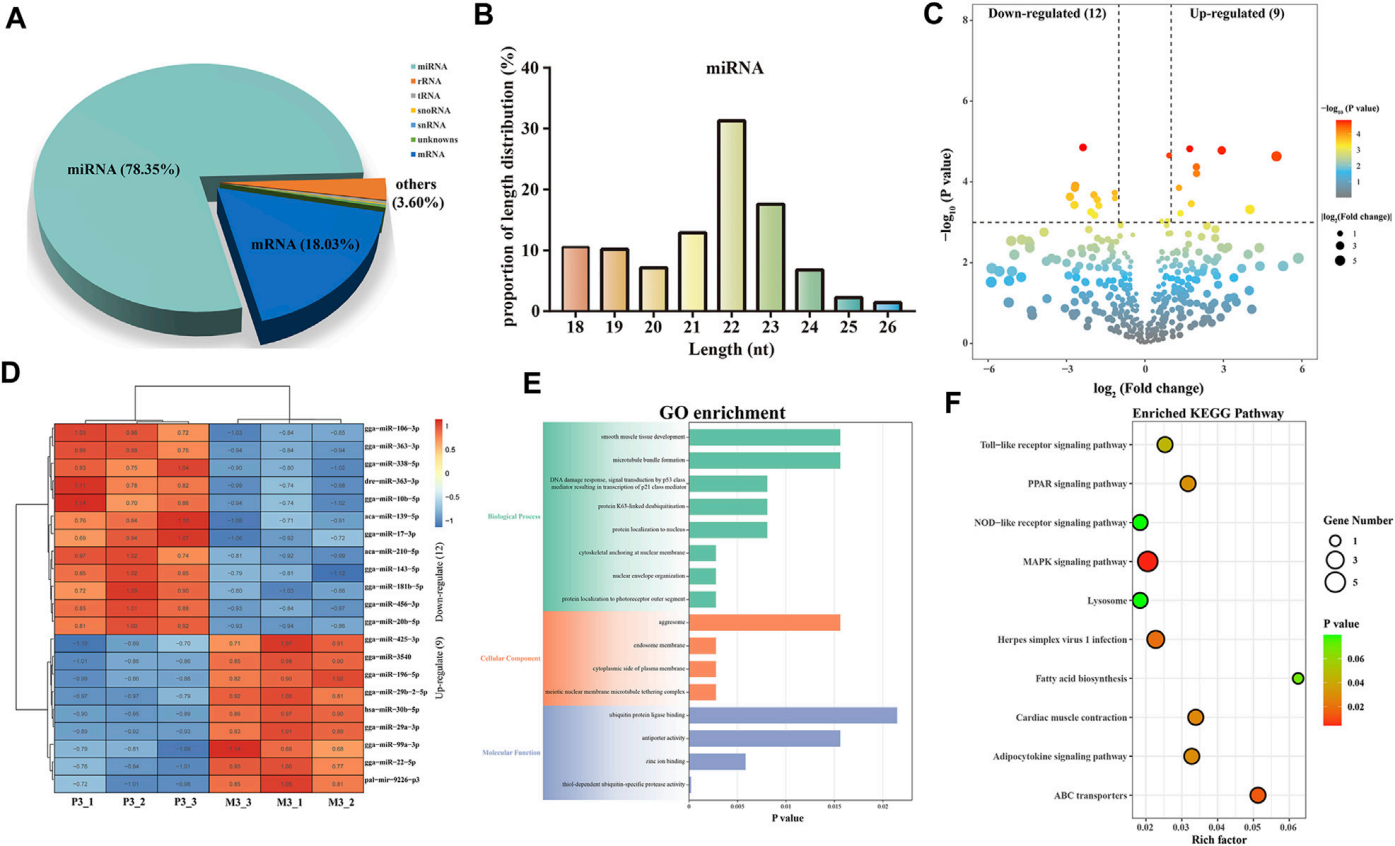
Identification and analysis of miRNAs associated with skeletal muscle maturation. proportions of small RNA types **(A)**. Length distribution of all miRNAs **(B)**. Volcano plot **(C)** and heatmap **(D)** of DEMIs. GO enrichment plot **(E)** and KEGG enrichment plot **(F)** of DEMs. The GO enrichment plot showed the top 8 most significant biological processes, the top 4 most significant cellular components, and the top 4 most significant molecular functions. The KEGG enrichment plot showed the top 10 most significant KEGG pathways.

In small RNA sequencing, we identified 78.35% miRNAs, 18.03% mRNAs, and 3.60% other small RNAs, including rRNA, tRNA, snRNA, and snoRNA (Figure 4A). By aligning with the precursor and genomic sequences, we identified 854 miRNAs, with the most abundant ones having a length of 22 bp, accounting for 31.26% (Figure 4B). Furthermore, we obtained 21 DEMIs, including 9 upregulated and 12 downregulated miRNAs (Figure 4C; Supplementary Table S6). These DEMIs exhibited good reproducibility within the group (Figure 4D). We then used TargetScan and miRanda to predict the targeting relationships between DEMIs and DEMs and constructed a DEMIs-DEMs network containing 21 DEMIs targeting 100 DEMs using Cytoscape (Supplementary Figure S1). To further explore the potential functions of DEMIs, we performed GO and KEGG enrichment analyses on the targeted DEMs.

The GO analysis revealed significant enrichment of terms closely related to skeletal muscle maturation, such as cytoskeletal anchoring at the nuclear membrane, smooth muscle tissue development, and meiotic nuclear membrane microtubule tethering complex. These findings suggest that DEMIs may regulate skeletal muscle maturation through the assembly and regulation of the cell cytoskeleton, smooth muscle tissue development, and control of cell division (Figure 4E; Supplementary Table S7). Furthermore, in the KEGG enrichment analysis, the MAPK signaling pathway, ABC transporters, PPAR signaling pathway, and fatty acid biosynthesis were found to be involved in the regulation of skeletal muscle maturation. These pathways collectively regulate cell proliferation, growth, energy balance, lipid metabolism, and related cellular signal transduction processes (Figure 4F; Supplementary Table S8). They play crucial roles in the development and maturation of skeletal muscle cells.

### 3.5 Identification and analysis of DELs associated with skeletal muscle maturation

**FIGURE 5:**
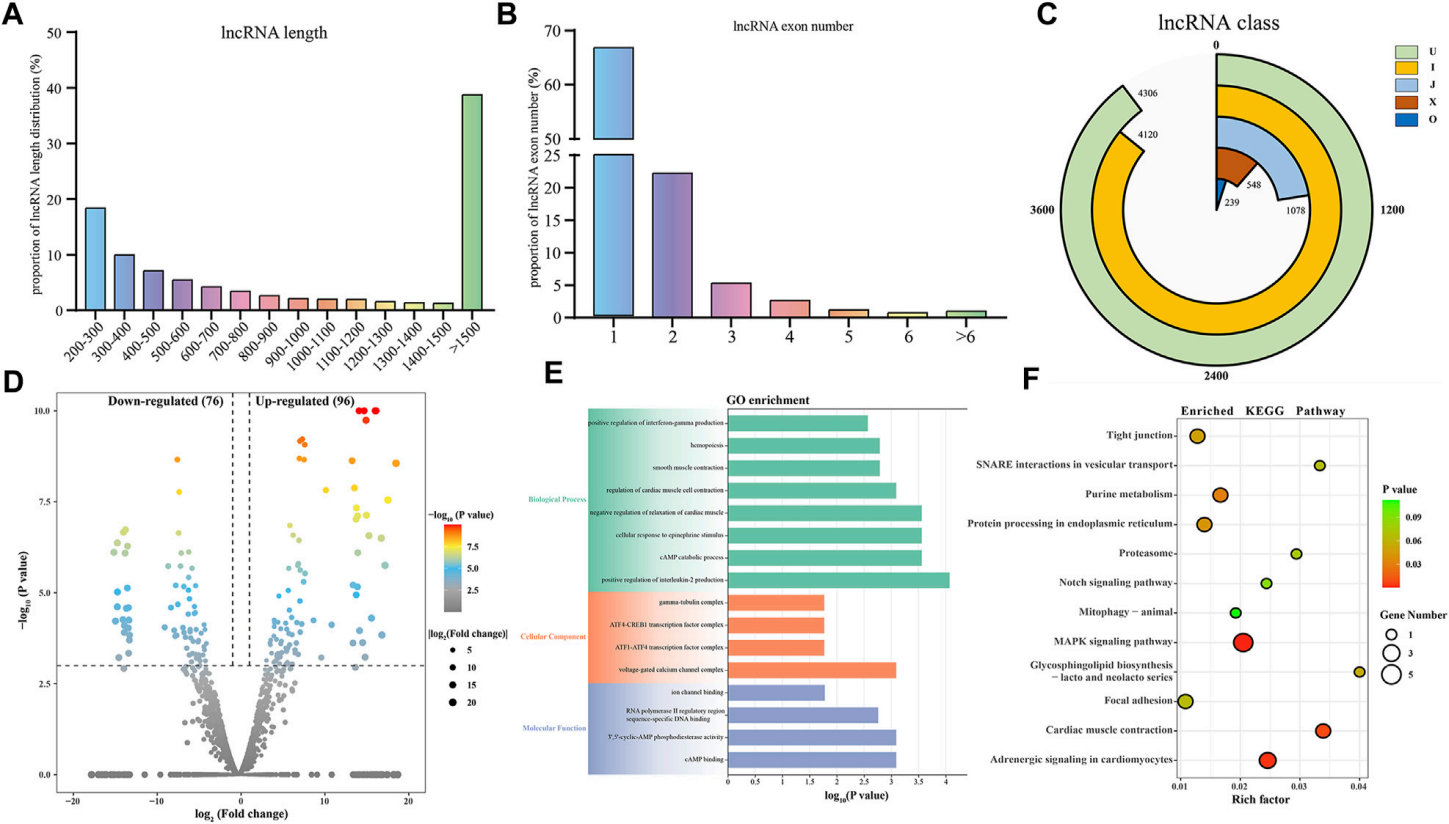
Identification and analysis of lncRNAs associated with skeletal muscle maturation. Proportion of lncRNA length distribution **(A)** and lncRNA exon number distribution **(B)**. LncRNA classification **(C)**. J: potentially novel isoform (fragment), which at least one splice junction is shared with a reference transcript. I: a transfrag falling entirely within a reference intron. O: generic exonic overlap with a reference transcript. X: exonic overlap with reference on the opposite strand. U: unknown, intergenic transcript. Volcano plot **(D)** of DELs. GO enrichment plot **(E)** and KEGG enrichment plot **(F)** of DEMs. The GO enrichment plot showed the top 8 most significant biological processes, the top 4 most significant cellular components, and the top 4 most significant molecular functions. The KEGG enrichment plot showed the top 10 most significant KEGG pathways.

In this study, we identified a total of 10,291 lncRNAs from the sequencing results of six samples. The length distribution of the identified lncRNAs is shown in Figure 5A, with the majority of lncRNAs being over 1500 bp in length. Among the identified lncRNAs, 66.83% had only one exon, while 22.22% contained two exons (Figure 5B). Furthermore, based on the class code for coding potential prediction, we categorized the identified lncRNAs, and apart from unknown intergenic transcripts, the most common category consisted of 4,120 lncRNAs falling entirely within intronic regions (Figure 5C). We identified a total of 172 DELs, including 77 downregulated and 97 up-regulated lncRNAs. To explore the functions of lncRNAs, we predicted their cis-regulated target genes (Figure 5D; Supplementary Table S9). lncRNAs may play a cis-regulatory role on adjacent target genes. In this study, the python script was used to predict the upstream and downstream 100,000 coding genes. Subsequently, GO and KEGG enrichment analyses were performed on these cis-regulated genes, revealing a potential correlation of DELs with pathways such as the MAPK signaling pathway, tight junction, smooth muscle contraction, and cellular response to epinephrine stimulus. These enrichment analysis results indicate the potential involvement of DELs in the regulation of these signaling pathways and cellular processes (Figures 5E, F; Supplementary Tables S10, 11). These findings suggest that DELs may play a role in skeletal muscle development and maturation, associated with these critical cellular processes and signaling pathways. Finally, we constructed a targeting network containing 21 miRNAs and 102 lncRNAs (Supplementary Figure S2).

### 3.6 Construction of lncRNA-miRNA-mRNA regulatory network

**FIGURE 6:**
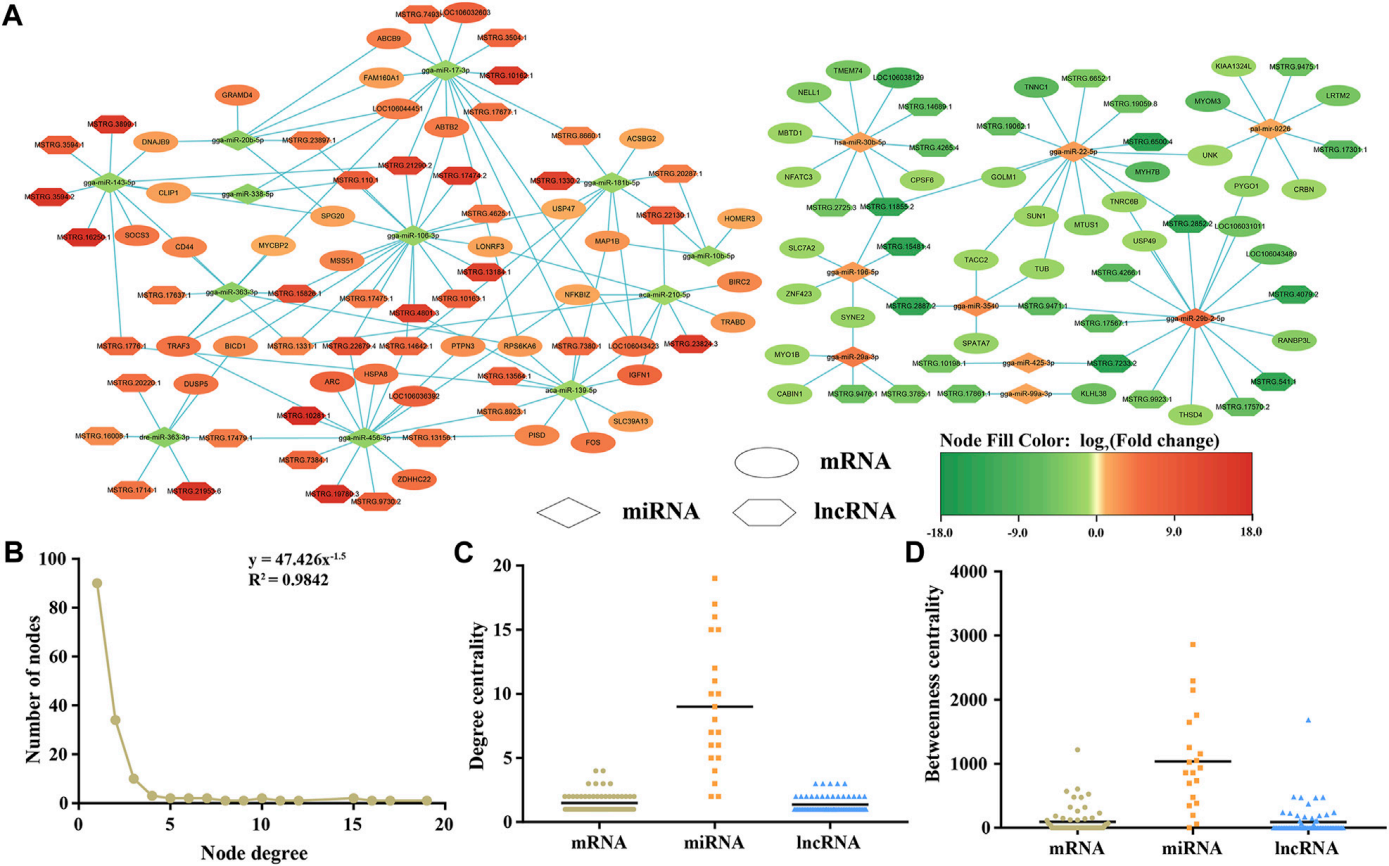
An overview the ceRNA regulatory network related to skeletal muscle maturation. lncRNA–miRNA–mRNA network **(A)**. Hexagon represents lncRNAs, diamond represents miRNAs, ellipse represents mRNAs, green is downregulated, and red is upregulated. Degree distribution of the ceRNA network **(B)**. The degree centrality difference among mRNAs, miRNAs and lncRNAs **(C)**. The betweenness centrality difference among mRNAs, miRNAs and lncRNAs **(D)**.

Based on the predicted target relationships of the DELs, DEMs, and DEMIs, as well as the ceRNA theory, we constructed a lncRNA-miRNA-mRNA regulatory network for the maturation of leg skeletal muscle (Figure 6A). This ceRNA network consists of 102 lncRNAs, 21 miRNAs, and 100 mRNAs. Further analysis of the ceRNA network revealed a power-law distribution of node degrees with a slope of −1.5 and an R^2^ value of 0.9842 (Figure 6B), indicating typical scale-free characteristics of a biological network. Additionally, when comparing lncRNAs and mRNAs, miRNAs exhibited distinct centrality and degree centrality, further emphasizing their crucial roles in this ceRNA network (Figures 6C, D).

**FIGURE 7:**
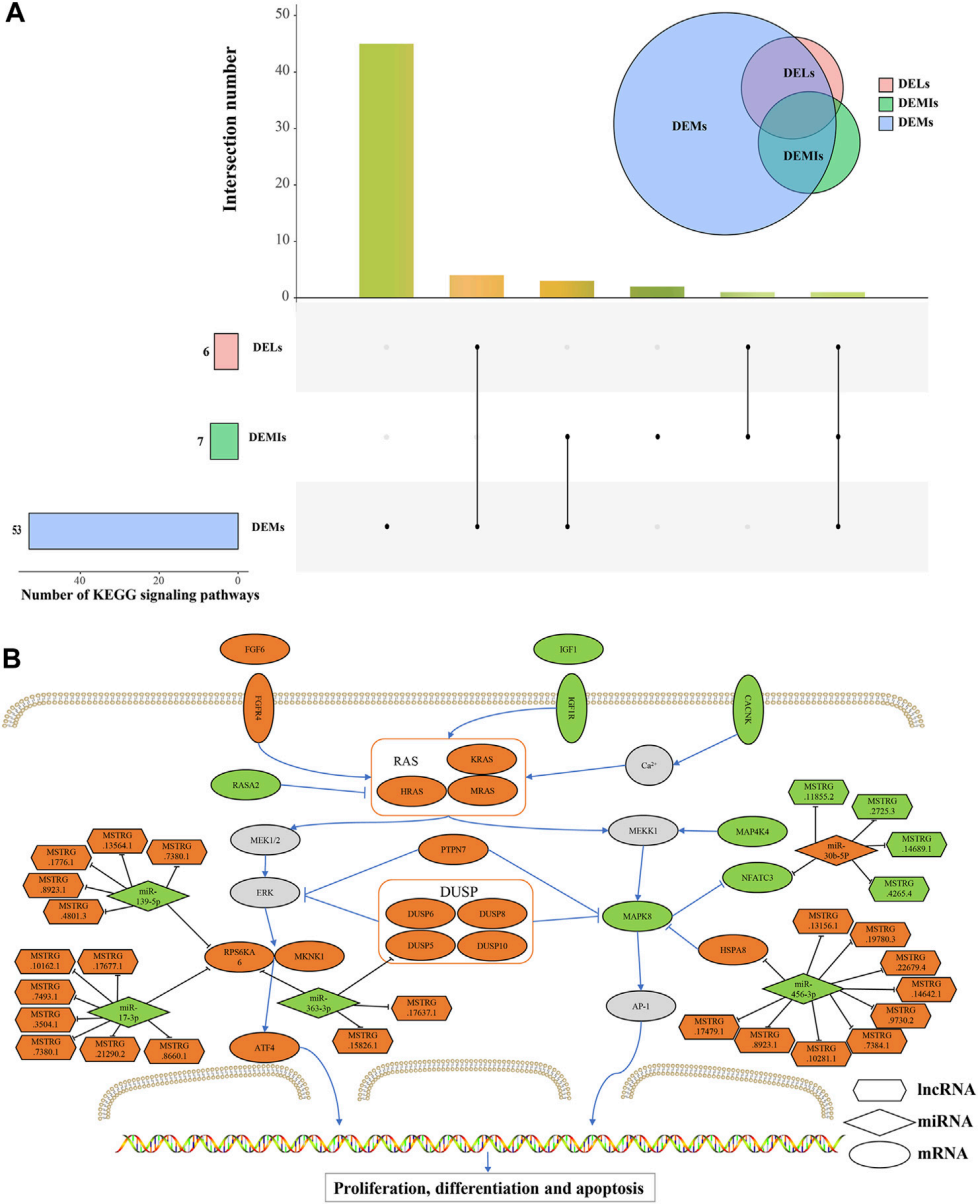
Schematic diagram of MAPK signaling pathway network related to skeletal muscle maturation in Magang goose. Upset diagram of the signaling pathways obtained from the enrichment analysis of DEMs, cis-regulatory genes of DELs, and target DEMs of DEMIs **(A)**. Schematic diagram of MAPK signaling pathway network **(B)**. Hexagons represent lncRNAs, diamonds represent miRNAs, ellipses represent mRNAs, and the orange color represents upregulation, while the green color represents downregulation.

Furthermore, we performed an intersection of the signaling pathways obtained from the enrichment analysis of DEMs, cis-regulatory genes of DELs, and target DEMs of DEMIs. As a result, we found that only the MAPK signaling pathway was co-enriched, revealing its potential critical role in the maturation process of Magang goose skeletal muscles (Figure 7A). The MAPK pathway is known to regulate muscle growth and hypertrophy by controlling the balance between muscle protein synthesis and degradation, ultimately influencing muscle size and development. Finally, based on the regulatory relationships among DEMs, DELs, and DEMIs, we constructed a schematic diagram of the MAPK signaling pathway network related to Magang goose skeletal muscle maturation (Figure 7B).

## 4 Discussion

The leg muscles of geese are the main components of their skeletal muscles, and the production of goose meat is closely related to the growth and development of skeletal muscles (Pan et al., 2022). In this study, we investigated the regulatory mechanisms underlying skeletal muscle maturation by collecting samples of leg skeletal muscles from Magang geese at 3 days and 3 months of age. Postnatal growth of skeletal muscles primarily occurs through an increase in the length and diameter of muscle fibers. Fiber hypertrophy after birth is associated with satellite cell proliferation and the accumulation of muscle-specific proteins, both positively correlated with fiber thickness and muscle mass (McFarland, 1999). Histological observations revealed that in the 3-month age group, the muscle fibers were arranged more densely, had larger diameter and area, and exhibited lower fiber density. Additionally, satellite cells showed a gradual migration towards the periphery, indicating a clear muscle fiber maturation. Additionally, RNA serves not only as a messenger between genes and proteins but also possesses various functions. Through transcriptome analysis, we identified 19,866 candidate mRNAs, 854 candidate miRNAs, and 10,291 candidate lncRNAs in the leg muscles of Magang geese. From a perspective on gene structure analysis, candidate lncRNAs are shorter compared to mRNAs, consistent with chicken expression profiles (Xu et al., 2019; Li et al., 2021). It is worth noting that mRNA levels, along with lncRNA and miRNA expression, decrease during skeletal muscle development, possibly due to functional differences at different stages of growth. Furthermore, the length distribution of miRNAs in the leg muscle of Magang geese followed a typical pattern, with a peak at 22 nt, consistent with other poultry species (Zhou et al., 2022).

Considering the differential expression of RNA between these two stages, these RNAs may be associated with skeletal muscle maturation. In particular, the thousands of non-coding RNAs exhibit cell type and tissue-specific expression, suggesting their potential roles in muscle development. In this study, we identified 1,949 DEMs, 21 DEMIs, and 172 DELs in these two stages, indicating their significant involvement in the maturation process of skeletal muscle. The development of skeletal muscle is a complex process that requires the coordinated regulation of multiple genes. We conducted KEGG enrichment analysis to further explore the potential molecular mechanisms by which DEMs regulate Magang Goose skeletal muscle development. Signaling pathways significantly enriched in skeletal muscle growth and development were classified into four potential functional categories: extracellular matrix and cell interactions; cytoskeleton, cell proliferation, and muscle protein metabolism; metabolic regulation; and signaling and gene regulation. It is well-known that skeletal muscle is the main site for glycogen storage, insulin-mediated glucose utilization, lipid metabolism, and fatty acid oxidation (Fan et al., 2017). Therefore, we also identified four metabolic related signaling pathways, namely, fatty acid metabolism, PPAR signaling pathway, adipocytokine signaling pathway, and insulin signaling pathway. With the development of skeletal muscle, intramuscular fat deposition increases. The activation of adipocytokine signaling, PPAR signaling, fatty acid metabolism, and fatty acid degradation pathways may regulate the accelerated deposition of intramuscular fat. Furthermore, previous studies have shown that the PPAR signaling pathway plays an important role in muscle growth and differentiation and is closely associated with skeletal muscle plasticity and dysfunction (Manickam and Wahli, 2017). In terms of extracellular matrix and cell interactions, the ECM is involved in the developmental process of skeletal muscle from embryonic stages to aging, not only maintaining the morphology of skeletal muscle but also participating in physiological functions such as myoblast migration, adhesion, proliferation, differentiation, and myotube formation (Zhang et al., 2021b). Similarly, tight junctions can participate in and control cell proliferation and gene expression, while serving as a cell structure that restricts the free passage of ions, proteins, and other molecules, which is crucial for the development of multicellular organisms (Yin et al., 2021). The growth of muscle tissue is primarily accomplished through two fundamental biological processes: cell proliferation and protein accumulation. Additionally, it has been found that activation of the canonical Wnt signaling pathway can induce muscle fiber hypertrophy (Von Maltzahn et al., 2012). In fact, previous studies have demonstrated that signaling pathways such as the MAPK signaling pathway (Keren et al., 2006), FoxO signaling pathway (Sanchez et al., 2014), and calcium signaling pathway (Al-Shanti and Stewart, 2009) play important roles in skeletal muscle growth and development, both in terms of signal transduction and gene regulation. These signaling pathways have complex and diverse relationships with skeletal muscle growth and development, but all have significant impacts on muscle growth and development. Interestingly, we observed that the MAPK signaling pathway was the only pathway enriched across DEMs, cis-regulatory genes of DELs, and target DEMs of DEMIs. This finding suggests its potentially more significant role in skeletal muscle maturation.

In goose skeletal muscle maturation after hatching, the MAPK signaling pathway plays a key role. The MAPK signaling pathway is a key intracellular signaling cascade that includes branches such as ERK (Extracellular Signal-Regulated Kinase), JNK (c-Jun N-terminal Kinase), and p38 MAPK. These branches can interact with each other through multiple pathways during skeletal muscle maturation. After poultry hatching, the MAPK signaling pathway can influence skeletal muscle development by regulating muscle cell proliferation, differentiation, and maturation (van Wessel et al., 2010). In recent years, the ceRNA hypothesis has attracted widespread attention, and more and more studies have constructed ceRNA networks related to ncRNAs in skeletal muscle growth and development. In addition, Luo et al. conducted a functional network analysis of differentially expressed miRNAs and mRNAs between sex-linked dwarf chickens and normal chickens and found that let-7b and miR-128 may play a key role in GHR deficiency-induced muscle mass loss through the MAPK pathway (Luo et al., 2016). This study identified five DEMIs (miR-139-5p, miR-17-3p, miR-363-3p, miR-456-3p, and miR-30b-5p) that may play a significant role in regulating the MAPK signaling pathway during the maturation process of the Magang goose leg muscle by targeting four DEMs (*RPS6KA6*, *DUSP5*, *HSPA8* and *NFATc3*). It is worth noting that among these, DEMIs and some DEMs have been shown to play important roles in skeletal muscle growth and development through related research.

*IGF1* (Insulin-like Growth Factor 1) and *FGF6* (Fibroblast Growth Factor 6) are growth factors that play critical roles in muscle development and growth. *IGF-1* can regulate protein synthesis and degradation pathways, and changes in *IGF-1* signaling in skeletal muscle can significantly impact muscle fiber size and function (Yoshida and Delafontaine, 2020). At the age of 3 days, goose muscle development may be in a more active and rapid stage, so the expression levels of *IGF-1* and *IGF1R* may be relatively high. Additionally, the upregulation of *FGF6* and its receptor *FGFR4* can stimulate muscle cell proliferation, migration, and muscle differentiation (Armand et al., 2006). The differential expression levels of these two growth factors also suggest their different regulatory roles during the rapid proliferation, differentiation, and maturation processes of muscle cells. RAS is a class of GTPases that regulates downstream signaling by hydrolyzing and binding to GTP. In the MAPK signaling pathway, RAS acts as a key molecular switch, participating in the regulation of cell proliferation, differentiation, and survival, among other biological processes (Sundaram, 2006). PTP (Protein Tyrosine Phosphatase) and DUSP (Dual-Specificity Phosphatase) are phosphatases in the MAPK signaling pathway that regulate the activity of MAPK through dephosphorylation (Rony, 2004; Hepworth and Hinton, 2021). In addition, among the four inducible nuclear MKPs, *DUSP5* is unique and exhibits absolute specificity for ERK1/2. Furthermore, growth factor-induced expression of *DUSP5* is mediated by ERK activity, making it a classic negative feedback regulator of this signaling pathway. *DUSP5* also tightly binds to its substrate and can anchor inactivated ERK in the nucleus. *DUSP5* has also been shown to regulate cardiac fibroblast proliferation and cardiac hypertrophy (Seternes et al., 2019). Through targeting relationship prediction, we found that DUSP5 had a targeting relationship with miR-363-3p. Studies have shown that miR-363 is related to the growth of developing limbs, and miR-363 was found to be expressed in wing buds and leg buds during early development. Meanwhile, miR-363 was found to be a negative regulator of adipogenesis in adipose-derived stromal cells (Darnell et al., 2006; Liu et al., 2016). The downregulation of miR-363-3p may promote fat deposition in skeletal muscle of geese. Interestingly, miR-363-3p is also predicted to target *RPS6KA6* (Ribosomal Protein S6 Kinase A6), which is believed to be a component of the IGF-1/AKT/mTOR signaling pathway and plays a key role in skeletal muscle formation (Xu et al., 2020). At the same time, it was found that the expression level of *RPS6KA6* was significantly increased in the skeletal muscle of patients with muscular dystrophy (Mamoor, 2022). In addition, *RPS6KA6* has a potential targeting relationship with miR-139-5p and miR-17-3p. The expression of miR-139-5p was upregulated during proliferation of C2C12 myoblasts, but showed an opposite trend during differentiation, which could slow down the growth of myoblasts and inhibit differentiation (Xu et al., 2020). miR-17 was found to effectively promote the differentiation of C2C12 myoblasts, acting on *Ccnd2*, *Jak1*, and *Rhoc* genes, and is essential for cell proliferation and/or fusion (Kong et al., 2019). Additionally, *ATF4* (Activating Transcription Factor 4) belongs to the activating transcription factor family, which can bind to DNA sequences and activate or suppress gene expression. It plays an important role in the regulation of muscle atrophy in skeletal muscle (Ebert et al., 2022). Under the regulation of ncRNA and mRNA through the ERK signaling pathway, the differentiation ability in the leg muscle of geese aged 3 months was significantly decreased compared with that of geese aged 3 days.

On the other hands, under the regulation of *PTPN7* and *DUSP*, *MAPK8* showed a downregulation trend. *MAPK8* is a member of the MAPK signaling pathway involved in many biological and molecular processes such as cell proliferation, differentiation, and apoptosis. HSPs (heat shock proteins) are a conserved family of molecular chaperones involved in regulating skeletal muscle plasticity. *HSPA8* is a member of the HSP70 family, and upregulation of HSP70 helps maintain muscle fiber integrity and promotes muscle regeneration and recovery. Conversely, during muscle inactivity and aging, the expression of HSP70 decreases. Furthermore, the loss of *HSPA8* has been identified as a key mechanism leading to muscle atrophy, impaired contractile function, and reduced regenerative capacity (Senf, 2013). HSPA8 is regulated by miR-456-3p, miR-456-3p is expressed in skeletal muscle, and more studies are needed to understand its specific function. NFATC3 is regulated by MAPK8, and calcium-dependent phosphatase signaling mediated by NFATc3 is critical in early myogenic differentiation events and may also help regulate fiber type conversion during early skeletal muscle development in ducks (Delling et al., 2000; Shu et al., 2015). At the same time, NFATC3 was regulated by miR-30b-5p and 4 DELs. The miR-30 family is associated with muscle development and protein synthesis. Many functions of the miR-30 family have been studied, including regulating fibrosis, apoptosis, and hypertrophy in cardiac muscle cells. Overexpression of miRNAs from the miR-30 family promotes differentiation, while inhibition limits differentiation of myoblasts *in vitro* (Guess et al., 2015). Liang et al. found that lncRNA AK017368-miR-30c-containing-6A acts as a ceRNA, and (Liang et al., 2018) promotes myoblast proliferation and inhibits differentiation by attenuating the function of miR-30c (Liang et al., 2018). lncRNAs extensively participate in the regulation of skeletal muscle growth and development through the ceRNA mechanism. lncRNAs primarily function as sponges to absorb miRNAs. For example, lncIRS1 acts as a sponge for the miR-15 family, regulating the expression of insulin receptor substrate 1 and thereby promoting skeletal muscle myogenesis while controlling muscle atrophy (Li et al., 2019). The lncRNA-miR-1611-SIXI ceRNA axis can influence the proliferation and differentiation of myoblasts and participate in the conversion of skeletal muscle fiber types (Ma et al., 2018). In this study, we predicted that 29 DELs may regulate the maturation of goose skeletal muscle by modulating the MAPK signaling pathway through the ceRNA mechanism. However, the roles of these DELs in the growth and development of goose skeletal muscle have not been studied and require further investigation.

## 5 Conclusion

In conclusion, by analyzing mRNA, miRNA and lncRNA in the maturation stage of skeletal muscle in the leg muscles of Magang geese, this study found that29 lncRNAs, 5 miRNAs, and 4 mRNAs may play a key role in the maturation of skeletal muscle through MAPK signaling pathway. These contributes to a deeper understanding of the molecular mechanisms underlying Magang geese skeletal muscle maturation and provides valuable references for future research in this field.

## Data Availability

The datasets presented in this study can be found in online repositories. The names of the repository/repositories and accession number(s) can be found in the article/Supplementary Material.
