# Supplementary material for: Non-coding RNA regulation of Magang geese skeletal muscle maturation via the MAPK signaling pathway
Source: Front Physiol. 2024 Jan 19;14:1331974. doi: 10.3389/fphys.2023.1331974 (PMC10834734; doi:10.3389/fphys.2023.1331974)
Supplement: Supplementary file 1 [file DataSheet1.ZIP › Supplement/Figure supplement.docx]

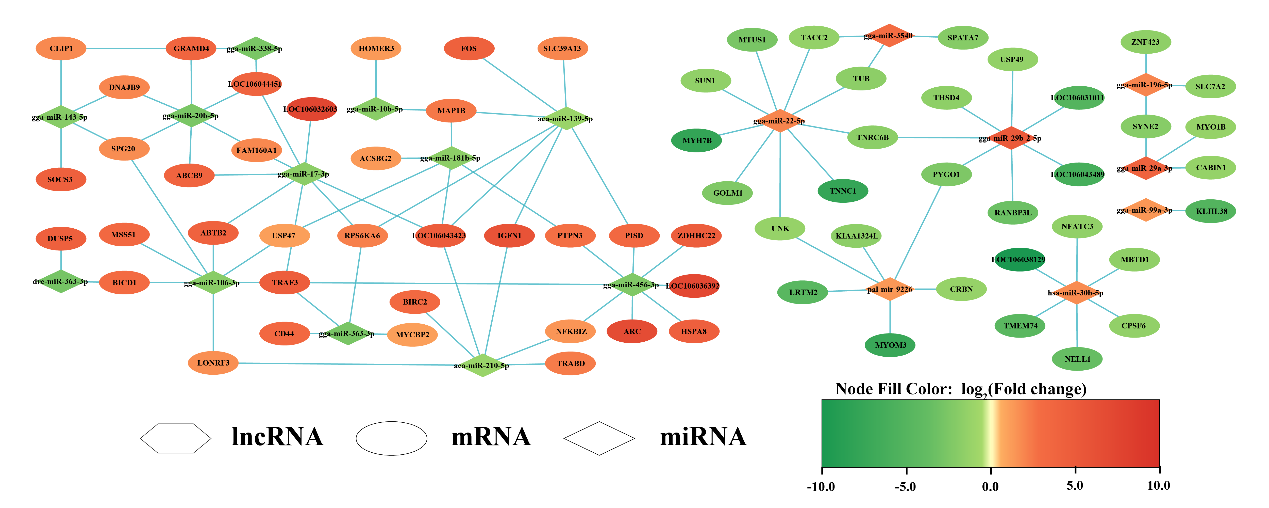


Figure S1: The DEMIs-DEMs targeted prediction network.


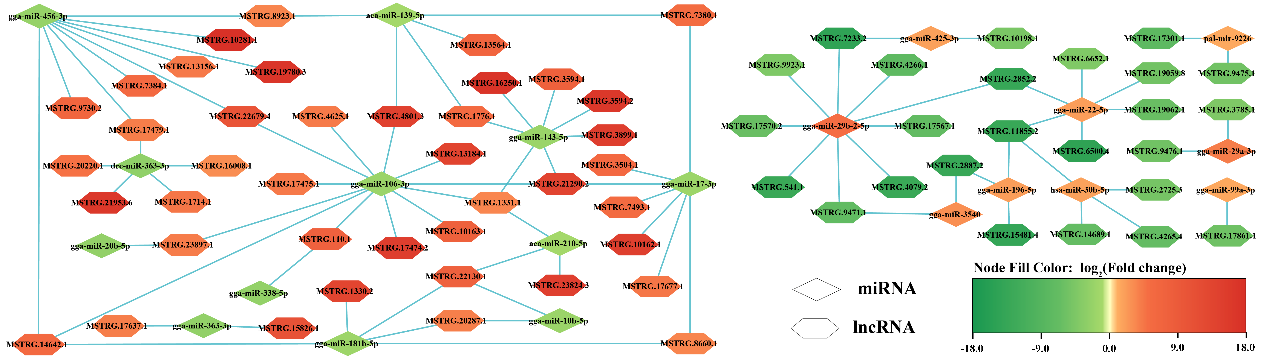


Figure S2: The DEMIs-DELs targeted prediction network.
